# The Prevalence of Chronic Kidney Disease in a Primary Care Setting: A Swiss Cross-Sectional Study

**DOI:** 10.1371/journal.pone.0067848

**Published:** 2013-07-03

**Authors:** Yuki Tomonaga, Lorenz Risch, Thomas D. Szucs, Patrice M. Ambuehl

**Affiliations:** 1 Institute of Social and Preventive Medicine, Medical Economics, University of Zurich, Zurich, Switzerland; 2 Labormedizinische Zentren Dr. Risch, Liebefeld, Switzerland; 3 Division of Clinical Biochemistry, Center of Chemistry and Biomedicine Innsbruck, Innsbruck Medical University, Innsbruck, Austria; 4 European Center of Pharmaceutical Medicine, University of Basel, Basel, Switzerland; 5 Stadtspital Waid, Renal Division, Zurich, Switzerland; University of KwaZulu-Natal, South Africa

## Abstract

Chronic kidney disease (CKD) often remains clinically silent and therefore undiagnosed until a progressed stage is reached. Our aim was to estimate the prevalence of CKD in a primary care setting in Switzerland. A multicenter, cross-sectional study with randomly selected general practitioners was performed. Adults visiting their general physician’s cabinet during defined periods were asked to participate. Baseline information was reported on a questionnaire, urine and blood samples were analyzed in a central laboratory. Renal status was assessed using the Kidney Disease: Improving Global Outcomes (KDIGO) classification. Extrapolation of results to national level was adjusted for age and gender. One thousand individuals (57% females) with a mean age of 57±17 years were included. Overall, 41% of the patients had normal estimated glomerular filtration rate (eGFR) and albumin creatinine ratio (ACR), whereas 36% of the subjects had slightly reduced excretory renal function with physiological albuminuria based on normal ACR. Almost one fourth of the subjects (23%) had either a substantially reduced eGFR or high levels of ACR. About 10% of the patients had a substantially reduced eGFR of <60 ml/min/1.73 m^2^, and 17% showed relevant proteinuria (ACR >30 mg/g creatinine). Extrapolation to national level suggests that about 18% of primary care patients may suffer from CKD. CKD prevalence in a primary care population is therefore high, and preventive interventions may be advisable, in particular as CKD prevalence is likely to rise over the next decades.

## Introduction

Chronic kidney disease (CKD), defined as renal damage with persistent and usually progressive deterioration of ultrafiltration, is a worldwide public health problem [1]. Several studies have shown that patients with CKD have increased risk of cardiovascular events and increased risk of death [2]–[12]. Moreover, the ageing of the population in western countries and the generally increasing rates of obesity, hypertension, and diabetes worldwide suggest that the incidence and prevalence of CKD will rise over the next decades [13], [14].

The classification of CKD is mainly based on measured or estimated glomerular filtration rate (GFR). In CKD stages 1 and 2, kidney function is normal (GFR >90 ml/min/1.73 m^2^) or slightly reduced (GFR 60–89 ml/min/1.73 m^2^), respectively, with evidence of renal damage (e.g. proteinuria). In CKD stages 3 and 4, functional impairment is moderate (GFR 30–59 ml/min/1.73 m^2^) or severe (GFR 15–29 ml/min/1.73 m^2^), respectively. Finally, CKD stage 5 is defined by kidney failure (GFR <15 ml/min/1.73 m^2^) or dialysis, and is also termed end-stage renal disease (ESRD) [15]. Recently, the Kidney Disease: Improving Global Outcomes (KDIGO) foundation performed a meta-analysis to investigate the relationship of estimated GFR (eGFR) and albuminuria with mortality and kidney outcomes: the results confirmed the current definition for CKD, i.e. GFR <60 ml/min/1.73 m^2^ or urinary albumin to creatinine ratio (ACR) >30 mg/g [16].

In the last decades, the majority of studies on CKD focused on its most advanced stages (stages 4–5). ESRD patients usually present with many complications, high mortality, strongly reduced quality of life, and high health care expenditures [17], [18]. At this stage renal replacement therapy (RRT), consisting in hemodialysis, peritoneal dialysis, hemofiltration, and kidney transplantation, becomes necessary [19].

Unfortunately these interventions are expensive and not always available. Hemodialysis costs amount to 530 Swiss Francs per session [20]. whereas renal transplantation costs are 58,300 Swiss Francs in the first year [21]. Moreover, there is an increasing gap between the number of donors and the number of patients waiting for a kidney: whereas the number of kidney transplantations between 2002 and 2011 slowly increased from 204 to 282 (i.e. +38%), the number of patients on the waiting list increased from 744 to 1,185 (i.e. +59%). In 2009 the mean waiting time for a donor organ was around 700 days [22].

Except for RRT, there is no other treatment for CKD patients with ESRD. Even at early stages, actual treatment options mainly aim to prevent or slow disease progression by controlling risk factors such as hypertension, diabetes, and obesity [23]. In short, prevention plays a key role in CKD management.

One of the biggest issues in CKD prevention is actually disease awareness. In the Kidney Early Evaluation Program (KEEP), a community-based screening program, only 10.0% of the 26,213 participants were aware of suffering from CKD. The proportion in awareness was particularly low for early CKD, with 5.1%, 6.3%, and 10.0% for stages 1 to 3, respectively. In contrast, almost 40% of the patients with CKD stage 4, and 60% of those with CKD stage 5 were aware of having renal disease [24]. Thus, despite the fact that effective preventive measures exist, many CKD patients remain undiagnosed and untreated. In this regard, family physicians play a fundamental role by timely diagnosis of diabetes mellitus and hypertension in their patients, the latter being the major contributors to CKD. However, screening for signs of renal damage is required, too. Early diagnosis and treatment of CKD and CKD related complications (e.g. anaemia, dyslipidemia, metabolic bone disease, metabolic acidosis, etc.) might prevent or slow the development of further sequelae and delay the requirement for RRT [25], [26]. In a large retrospective study including about 12,000 patients with stage 3 or 4 CKD in primary care, the authors reported that CKD management, especially without the involvement of a nephrologist, was not optimal: 72% of patients with diagnosed CKD lacked annual urine protein testing, 26% were not receiving appropriate angiotensin blockade, and 20% were taking potentially harmful drugs [27]. Moreover, whereas annual screening for anaemia was common (80%), annual testing for metabolic bone disease was less frequent (calcium 56%, vitamin D 26%, parathyroid hormone 13%).

One of the first steps to improve CKD management is knowledge about CKD prevalence. Consequently, in the last few years, research has focused on the epidemiology of CKD [28], [29]. Supposing that the simplest way to identify CKD is through a family doctor, the aim of this study was to estimate the prevalence of CKD in a primary care setting in Switzerland. The results of this study may provide important information for future national preventive programs, optimizing the resource allocation process. The estimations at national level may improve public awareness for CKD and CKD related diseases.

## Materials and Methods

### Study Design and Patient Population

A cross-sectional, multicentre, non-interventional study was conducted in seven of the 26 Swiss cantons, including all five Swiss cantons with university affiliated medical faculties (i.e. Basel, Bern, Geneva, Vaud, and Zurich), the largest canton in central Switzerland (Lucerne), and the Italian speaking canton of Ticino. The selected cantons were home to nearly 60% of the entire Swiss population in 2010 and represent all three major language regions in Switzerland (German: Basel, Bern, Lucerne, and Zurich; French: Geneva and Vaud; Italian: Ticino) [30]. Physicians invited to participate in the study were randomly selected from the total pool of general practitioners (GPs) in each canton. Random selection was performed by a computer program generating random numbers. Physicians from 33 offices agreed to participate. The study coordination centre defined the days of patient inclusion by the GPs meeting inclusion criteria (i.e. age ≥18 years and the ability to provide written inform consent). Emergency patients and patients for which the participation in the study might have caused relevant delays in patient management were excluded for ethical reasons. Otherwise, all patients were consecutively included into the study. The study was conducted according to the Declaration of Helsinki (as revised in 2008) and with the International Conference on Harmonization-Good Clinical Practice (ICH-GCP) standards. The study was approved by all seven cantonal ethics committees: Ethikkommission beider Basel (EKBB), Kantonale Ethikkommission Bern (KEK), Commission d’éthique pour la recherche clinique dans le Canton de Genève, Ethikkommission des Kantons Luzern, Comitato etico cantonale del Canton Ticino, Commission cantonale (VD) d’éthique de la recherche sur l’être humain, and Kantonale Ethikkommission (KEK) Zürich.

### Measures

Socio-demographic variables, clinical status and co-morbidities were reported on a questionnaire. Urine and blood samples were sent to a central laboratory for analysis. A spot urine was collected in a Greiner Vacuette tube without preservatives (Greiner Bio One, Krems, Austria), whereas venous blood was collected in Sarstedt Monovette EDTA tubes and in serum tubes containing separation gel (Sarstedt, Sevelen, Switzerland). After serum sample centrifugation, the samples were mailed to the central laboratory (*Labormedizinisches Zentrum Dr. Risch*) using overnight delivery service by the Swiss Postal Service. Laboratory analysis was performed on the day the samples were received. Laboratory parameters were determined on an Abbott ARCHITECT ci4100 analyzer platform (Abbott, Baar, Switzerland), a Sysmex XT-5000 hematology analyzer (Sysmex Digitana, Horgen, Switzerland), and a Bio-Rad D-10 HPLC system for the determination of glycated haemoglobin (HbA1c; Biorad, Pratteln, Switzerland). The following parameters were measured to assess kidney function and damage: serum and urinary creatinine using the Jaffé method, cystatin C in the serum and urinary albumin (all from Abbott, Baar, Switzerland). In our hands, the intra-assay coefficient of variation (CV; n = 20) for creatinine was 1.5% at 60 µmol/L, 1.0% at 168 µmol/L, and 0.7% at 624 µmol/L. The respective CV’s were 1.8% at 0.7 mg/L and 2.0% at 3.5 mg/L for cystatin C, and 1.6% at 32.5 mg/L, 1.5% at 119.5 mg/L for urinary albumin.

### Statistical Analysis

Data were analyzed with IBM SPSS® Statistics 19.0 and Microsoft Office Excel 2007. Chi-square tests and t-tests were used for categorical and continuous variables, respectively. A two-tailed p value <0.05 was considered statistically significant.

The eGFR was calculated with the CKD-EPI equation [31], [32]. All patients were stratified into CKD stages using the classification recently proposed by KDIGO [33].

Extrapolation of CKD prevalence in primary care to national level was based first on the 3,769,686 Swiss patients older than 15 years of age who had visited a GP at least once in 2007 (i.e. 62.7% of the Swiss population >15 years), as reported by the Swiss Federal Statistical Office [34]. Secondly, using the percentages calculated in our study sample and adjusting the results for age and gender, the prevalence of CKD patients in primary care in Switzerland was estimated.

Generalized linear models were fitted to control for factors that may be related to reduced eGFR or elevated ACR. In the first model, age, gender, and clinical characteristics of the study population were entered. In the second model, the laboratory parameters were analyzed. All variables showing significant results were used in a third model. The variables confirming significance in the third generalized linear model were finally combined in a simple linear regression analysis. The coefficient of determination (R square) and Pearson correlation coefficients were calculated. Significant variables were tested for multicollinearity by calculating the variance inflation factors (VIFs).

## Results

### Socio-demographic and Clinical Characteristics of the Study Population

Among the 1,000 individuals recruited, 57% were female, and the mean age was 57±17 years. The main socio-demographic and clinical characteristics of the patients according to gender are shown in Table 1. Gender comparisons revealed that males had a significantly higher BMI, higher systolic and diastolic blood pressures, a higher mean arterial pressure, and a lower heart rate. Concerning co-morbidities, males reported a significantly higher prevalence of hypertension, diabetes, and myocardial infarction. Only depression was significantly more frequent among women. No relevant differences were found regarding family history for cardiovascular disease, diabetes or CKD, which were positive in about 30%, 20% and 5–6% of the patients, respectively.

**Table 1:** Socio-demographic and clinical characteristics of the study population according to gender.

Clinical status	Females	Males	P
	Mean±SD or %	Mean±SD or %	
N	567	433	–
Age (years)	56±18	57±16	0.155
BMI (kg/m^2^)	27±6	28±4	0.010
Systolic blood pressure (mmHg)	133±20	138±18	<0.001
Diastolic blood pressure (mmHg)	79±11	83±13	<0.001
Mean arterial pressure (mmHg)	97±13	101±13	<0.001
Pulse pressure (mmHg)	54±18	55±17	0.334
Heart rate (bpm)	74±11	71±12	0.001
Smoker	18.9	16.4	0.311
Hypertension	29.1	38.6	0.001
Depression	15.7	7.9	<0.001
Diabetes	12.0	17.3	0.012
Myocardial infarction	2.7	6.6	0.003
Heart failure	3.5	5.8	0.062
Family history of Diabetes	21.9	18.8	0.133
Cardiovascular disease	31.4	29.9	0.221
Chronic kidney disease	6.6	4.5	0.109

BMI, body mass index; bpm, beats per minute; mmHg, millimetre of mercury; N, number of subjects; SD, standard deviation.

### Laboratory Parameters

The results of the laboratory analysis showed that mean values of many parameters were significantly different between males and females (i.e. serum creatinine, albumin in the urine, urinary neutrophil gelatinase-associated lipocalin (NGAL), cystatin C, total cholesterol, high-density lipoprotein (HDL), high sensitive troponin, folic acid, ferritin, chloride, inorganic phosphate, HbA1c, alanine and aspartate transaminase, gamma-glutamyl transpeptidase, bilirubin total, and albumin). However, the majority of the laboratory parameters were within normal range for both genders (Table 2). For both genders, elevated values were found for total cholesterol, fasting low-density lipoprotein (LDL), parathyroid hormone (PTH), and glycated hemoglobin (HbA1c). Females showed slightly elevated values of C-reactive protein (CRP) and inorganic phosphate, whereas males had slightly elevated alanine transaminase values.

**Table 2:** Laboratory parameters in the recruited patient population.

Laboratory parameter	Females	Males	P
	Mean [95% CI]	Mean [95% CI]	
***Kidney***			
Serum creatinine (µmol/l)	71.4 [69.8–73.0]	87.7 [85.1–90.3]	<0.001
Albumin in urine (mg/l)	19.5 [16.0–22.9]	53.9 [40.3–67.5]	<0.001
Urinary NGAL (ng/l)	64.9 [46.8–83.0]	29.1 [23.9–34.4]	0.001
Cystatin C (mg/l)	0.81 [0.79–0.83]	0.86 [0.83–0.89]	0.004
***Lipids***			
Total cholesterol (mmol/l)	5.64 [5.53–5.74]	5.39 [5.28–5.49]	0.001
HDL (mmol/l)	1.66 [1.62–1.69]	1.35 [1.32–1.38]	<0.001
Fasting LDL (mmol) *	3.36 [3.13–3.60]	3.29 [3.09–3.49]	0.636
Fasting triglycerides (mmol/l) *	1.45 [1.32–1.58]	1.70 [1.46–1.95]	0.066
***Inflammation***			
High sensitive CRP (mg/l)	5.24 [4.47–6.00]	4.33 [3.42–5.24]	0.134
***Heart disease***			
BNP (pg/ml)	53.75 [47.7–59.8]	61.91 [46.5–77.3]	0.289
High sensitive troponin (ng/l)	1.94 [1.45–2.43]	3.74 [2.74–4.75]	0.001
***Nutritional parameters***			
Vitamin B12 (pg/ml)	292.6 [272.2–313.0]	289.8 [265.2–314.3]	0.858
Folic acid (mg/ml)	19.5 [18.6–20.4]	16.8 [15.9–17.6]	<0.001
Ferritin (ng/ml)	92.9 [81.5–104.4]	194.3 [177.9–210.7]	<0.001
***Electrolytes***			
Sodium (mmol/l)	141.8 [141.5–142.1]	141.7 [141.3–142.0]	0.580
Potassium (mmol/l)	4.82 [4.72–4.92]	4.75 [4.65–4.85]	0.330
Chloride (mmol/l)	103.3 [103.0–103.7]	102.6 [102.2–103.0]	0.004
***Calcium phosphate metabolism***			
Parathormone (pmol/l)	7.40 [7.01–7.79]	7.03 [6.54–7.52]	0.237
Calcium (mmol/l)	2.40 [2.39–2.41]	2.41 [2.39–2.42]	0.461
Inorganic phosphate (mmol/l)	1.70 [1.61–1.79]	1.51 [1.41–1.62]	0.009
***Diabetes mellitus***			
HbA1c (%)	5.93 [5.85–6.02]	6.09 [5.98–6.19]	0.023
***Liver function panel***			
Alanine transaminase (U/l)	26.2 [24.7–27.7]	39.3 [36.7–41.8]	<0.001
Aspartate transaminase (U/l)	26.7 [25.6–27.8]	31.7 [30.4–33.0]	<0.001
Gamma-glutamyl transpeptidase (U/l)	30.3 [26.4–34.1]	53.9 [47.0–60.7]	<0.001
Alkaline phosphatase (U/l)	72.9 [71.0–74.9]	72.3 [69.9–74.8]	0.701
Bilirubin total (µmol/l)	9.0 [8.6–9.4]	12.1 [11.5–12.7]	<0.001
Albumin (g/l)	44.1 [43.9–44.3]	45.0 [44.3–44.7]	<0.001

BNP, brain natriuretic peptide; CI, confidence interval; CRP, C-reactive protein; HbA1c, glycated haemoglobin A1c; HDL, high-density lipoprotein; LDL, low-density lipoprotein; NGAL, neutrophil gelatinase-associated lipocalin. * N = 107 for females and 100 for males.

### CKD Prevalence in the Study Population

**Figure 1:**
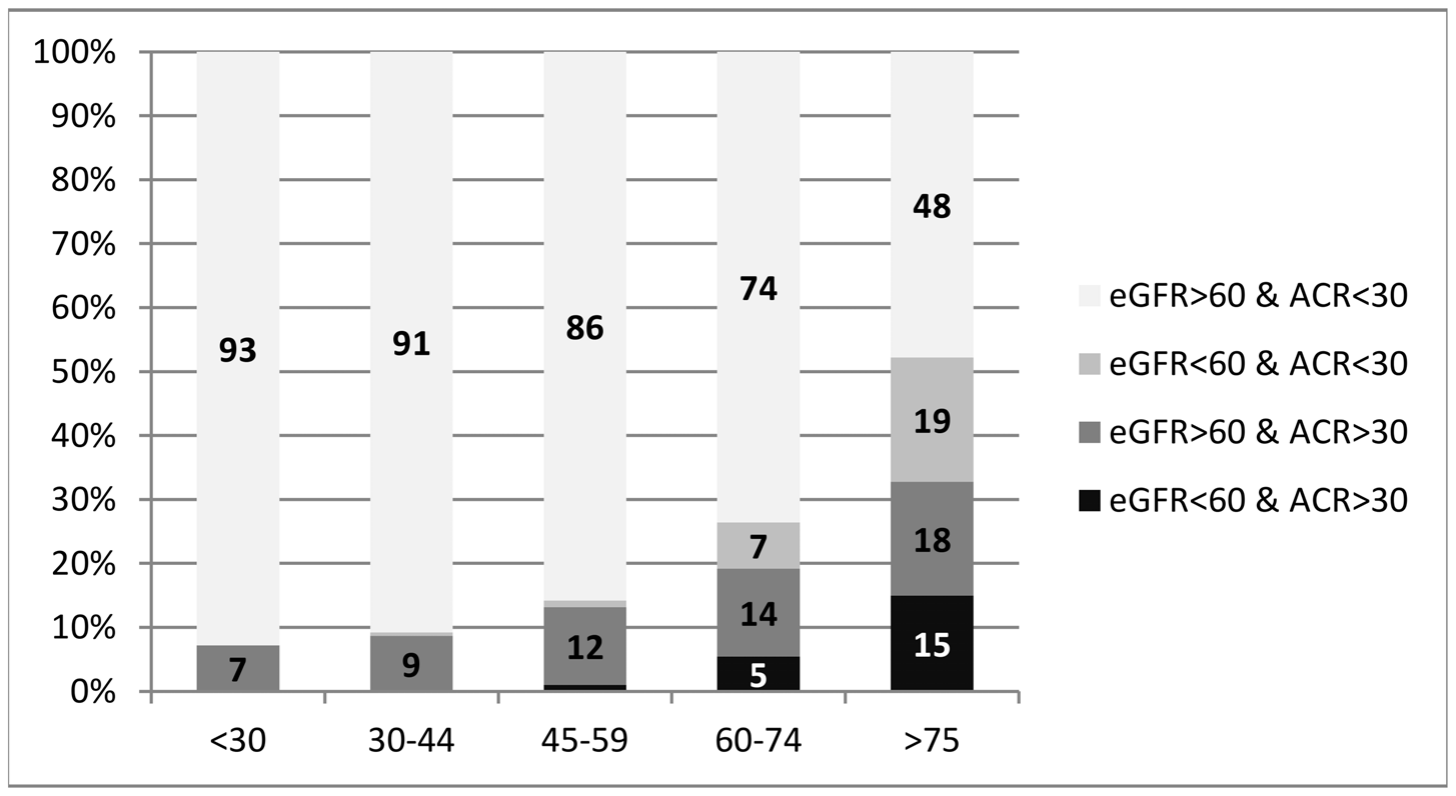
Percentage of patients with reduced eGFR and/or elevated ACR for different age groups. ACR, albumin-creatinine ratio (given as mg/mmol); eGFR, estimated glomerular filtration rate (given as ml/min/1.73 m^2^).

Overall, 41.1% of the patients had normal eGFR and ACR, whereas 35.9% of the subjects had slightly reduced excretory renal function (eGFR: 60–90 ml/min/1.73 m^2^) with physiological albuminuria based on normal ACR (<30 mg/g). About one tenth of the patients had a substantially reduced eGFR of <60 ml/min/1.73 m^2^, and 17.1% showed relevant proteinuria (ACR ≥30 mg/g). Almost one fourth of the analyzed subjects had CKD, i.e. they had either a substantial reduction in renal function or high levels of proteinuria (Table 3). CKD prevalence in our study population was clearly associated with increasing age: below 60 years of age CKD prevalence showed a slow increase (from 7% to 14%). Thereafter it increased faster, reaching 26% for patients aged 60–74 years and 52% for patients over 75 years of age (p<0.001; Figure 1). If compared to patients without renal disease, CKD patients showed a more balanced gender distribution (51.3% vs. 58.3% females, p = 0.060), but were significantly older (67±16 vs. 53±16 years, p<0.001), and had significantly higher BMI (28±5 vs. 27±5, p = 0.001). Moreover they showed a significantly higher prevalence of diabetes (28.3% vs. 10.1%, p<0.001), hypertension (53.9% vs. 27.0%, p<0.001), myocardial infarction (8.7% vs. 3.0%, p<0.001), and heart failure (10.9% vs. 2.6%, p<0.001). No significant differences were found concerning family history of diabetes, cardiovascular diseases, and CKD.

**Table 3:** Chronic kidney disease stages, as proposed in the KDIGO classification.

	Albuminuria stage			
EGFR Stage	A1 (<10 mg/g)	A1 (10–29 mg/g)	A2–3 (≥30 mg/g)	All
(ml/min/1.73 m^2^)	N (%)	N (%)	N (%)	N (%)
G1 (>105)	129 (12.9)	60 (6.0)	27 (2.7)	216 (21.6)
G1 (90–105)	156 (15.6)	66 (6.6)	33 (3.3)	255 (25.5)
G2 (75–89)	144 (14.4)	99 (9.9)	33 (3.3)	276 (27.6)
G2 (60–74)	59 (5.9)	57 (5.7)	33 (3.3)	149 (14.9)
G3–5 (<60)	25 (2.5)	34 (3.4)	45 (4.5)	104 (10.4)
All	513 (51.3)	316 (31.6)	171 (17.1)	1000 (100)

EGFR, estimated glomerular filtration rate (calculated using the CKD-EPI formula); KDIGO, Kidney Disease, Improving Global Outcomes.

### Regression Analyses

In order to control for factors that may be related to eGFR reduction or to an ACR increase regression analysis was performed. Concerning eGFR, in the first generalized linear model, age (p<0.001), gender (p<0.001), and heart failure (p<0.001) were significantly and independently correlated with eGFR. In the second model, statistically significant correlations with eGFR were found for cystatin C (p<0.001), total cholesterol (p<0.001), HDL (p<0.001), high sensitive C-reactive protein (CRP, p = 0.033), brain natriuretic peptide (BNP, p = 0.007), folic acid (p = 0.001), sodium (p<0.001), inorganic phosphate (p<0.001), HbA1c (p<0.001), alanine transaminase (p = 0.001), and albumin (p = 0.024). In the third model combining all significant factors, gender, age, cystatin C, HDL, BNP, sodium, inorganic phosphate, HbA1c, and albumin remained significantly correlated with eGFR. The combination of the variables from the third model in a simple linear regression model confirmed a strong relationship with the eGFR (R = 0.839, adjusted R square = 0.701). Particularly strong correlations were found for age (Pearson correlation coefficient ρ = −0.648) and cystatin c (ρ = −0.667). No multicollinearity problems were found (Table 4).

**Table 4:** Spearman correlation coefficients (ρ) between eGFR/ACR and the significantly correlated variables.

eGFR			
Variable	ρ	P	VIF
Age	−0.648	<0.001	1.517
Gender	0.089	<0.001	1.231
Cystatin C	−0.667	<0.001	1.460
HDL	−0.043	0.012	1.253
BNP	−0.298	0.015	1.151
Sodium	−0.156	<0.001	1.111
Inorganic Phosphate	−0.256	<0.001	1.076
HbA1c	−0.229	0.017	1.145
Albumin	0.128	0.003	1.121
R = 0.839, adjusted R square = 0.701.			
**ACR**			
**Variable**	**ρ**	**P**	**VIF**
Gender	0.102	0.001	1.040
Heart rate	0.098	<0.001	1.023
Diabetes	0.169	0.049	1.545
Heart failure	0.121	0.028	1.270
Urinary NGAL	0.154	<0.001	1.027
Cystatin C	0.247	<0.001	1.213
BNP	0.322	<0.001	1.282
HbA1c	0.215	0.001	1.588

R = 0.439, adjusted R square = 0.186.

BNP, brain natriuretic peptide; HbA1c, glycated haemoglobin A1c; HDL, high-density lipoprotein; NGAL, neutrophil gelatinase-associated lipocalin; VIF, variance inflation factor.

For ACR, the first model showed significant correlations with gender (p = 0.019), age (p = 0.036), heart rate (p = 0.021), diabetes (p = 0.001), and heart failure (p = 0.016). In the second model, significant results were found for urinary NGAL (p = 0.001), cystatin C (p = 0.001), BNP (p<0.001), and HbA1c (p<0.001). After combining all significant factors in a third model, gender, heart rate, diabetes, heart failure, urinary NGAL, cystatin C, BNP, and HbA1c remained significantly and independently correlated with ACR. The combination of these variables in a linear regression analysis showed a weak relationship (R = 0.439, adjusted R square = 0.186). The highest Pearson correlation coefficients were found for BNP (ρ = 0.322), cystatin c (ρ = 0.247), and HbA1c (ρ = 0.215). Again, no multicollinearity problems were found (Table 4).

### Extrapolation to National Level

In the extrapolation of CKD prevalence to the national level we made the assumption, that about 60% of the Swiss population visit a primary care physician at least once yearly (Table 5). Of those, almost 19% (i.e. ca 700,000 patients, 11.4% of the subjects older than 15 years) may suffer from CKD, having a substantially reduced eGFR (<60 ml/min/1.73 m^2^) and/or relevant proteinuria (ACR ≥30 mg/g).

**Table 5:** Chronic kidney disease prevalence in primary care.

Age group	N in CH	With at least 1 GP visit	CKD (eGFR <60 or ACR ≥30)
15–24	944 947	530 348	39 758
25–34	948 865	483 491	54 666
35–44	1 217 255	638 988	46 409
45–54	1 064 447	610 875	84 456
55–64	895 114	601 024	113 482
65–74	610 651	475 489	138 218
75+	505 433	429 471	226 665
Total	6 186 712	3 769 686	703 655
%	100.0%	60.9%	11.4%

ACR, albumin-creatinine ratio (given as mg/mmol); CH, Switzerland; CKD, chronic kidney disease; eGFR, estimated glomerular filtration rate (given as ml/min/1.73 m^2^); GP, general practitioners.

## Discussion

This study shows that CKD prevalence and/or renal function impairment in the general Swiss population is considerably high. In our sample only about 40% of the patients had a normal renal function with an eGFR ≥90 ml/min/1.73 m^2^ and an ACR <30 mg/g. About one third of the subjects showed slightly reduced filtration rate (eGFR 60–89 ml/min/1.73 m^2^) with physiological ACR, whereas 23% of the patients fulfilled the criteria of CKD (i.e. eGFR <60 ml/min/1.73 m^2^ and/or ACR >30 mg/g), as defined by KDIGO [33].

Our study sample, which was derived from a primary care population, is nevertheless comparable to that of the Swiss Survey on Salt, and, to some extent, to the general Swiss population. In the Swiss Survey on Salt, a prospective, nationwide survey conducted in 2010–2011 with a random sample of 1,377 subjects, the mean age was 47.3 years, with 51.2% females, 17.3% current smokers, a mean BMI of 25.1 kg/m^2^, and a 25.6% prevalence of hypertension (32.3% and 19.1% for male and females respectively) [35]. At national level, the Swiss Federal Statistical Office reports the mean age of the adult patients visiting at least once a primary care physician in 2007 being 48.7 years (48.9% males) [34]. Moreover, the following prevalence were estimated within the Swiss population in 2007∶27.9% smokers (32.3% males, 23.6% females), 15.0% hypertension (15.9%, 14.1%), 8.0% depression (6.2%, 9.8%), 3.0% diabetes (3.5%, 2.5%), and 2.1% myocardial infarction (3.1%, 1.2%) [36].

By extrapolation to national level, and after adjustment for age and gender, the percentage of patients in primary care that may have CKD is high, with almost 19% of the primary care population having substantially reduced renal function and/or relevant proteinuria. These results, again, are comparable to those found in the Swiss Survey on Salt, with a reported prevalence of about 7.7% of the included population for CKD stage 3 or higher. In our study, 10.4% of the patients had an eGFR <60 ml/min/1.73 m^2^. The difference of almost 3% may be explained by diverse recruitment strategies: whereas in our study the subjects were recruited in a primary care setting, in the Swiss Survey on Salt, the participants were recruited using a list of randomly selected households from the major Swiss telecommunication company’s home phone directory. For each household, one person was randomly selected and invited to participate in the study. It is reasonable to suppose that this sample was not only younger if compared to our study population, but also healthier and therefore less likely to suffer from CKD and other diseases. In the US National Health and Nutrition Examination Survey (NHANES) 1999–2006, a representative cross-sectional national survey, 9,536 participants were interviewed at home and/or received standardized medical examination in mobile study centers [37]. The prevalence of CKD was 18.3% (9.1% for CKD stage 3–5). Again, these rates are comparable to our results.

The generalized linear regression models showed that both eGFR and ACR are strictly correlated with gender, cystatin C, BNP, and HbA1c. It is interesting to note that whereas eGFR was correlated with age and several blood/urine parameters (e.g. sodium, HDL, inorganic phosphate), ACR was age independent and directly correlated to diabetes and heart failure. These results emphasize the importance of ACR as screening and prognostic factor for young patients and for patients with diabetes and/or heart failure. For example, in the Kidney Early Evaluation Program (KEEP) Annual Data Report 2007, it has been shown that ACR is the predominant positive screening test for younger age groups: in KEEP, about 80% and 60% of the CKD patients aged 18–30 and 31–45 years, respectively, showed elevated ACR with normal eGFR. Even higher percentages were found in the NHANES cohort from 1999–2004 [38]. In a case control study including non-diabetic and non-hypertensive patients it has been found that elevated ACR was significantly higher in patients with systolic heart failure, if compared to matched controls [39].

Some limitations of the study have to be considered. Firstly, screened subjects were volunteers and therefore not necessarily representative for the overall primary care population in Switzerland. In this study, emergency patients were excluded for ethical reasons. Moreover, it is possible that patients visiting the GP with a relatively serious/painful disease tend to refuse to participate. This may have resulted in an underestimation of the true CKD prevalence. A second limitation concerns the use of a creatinine based estimation for renal filtration function. In the last years, many formulas have been developed to calculate eGFR: the Modification of Diet in Renal Disease (MDRD) formula, the Mayo Quadratic formula, and the CKD-EPI formula [32], [40]–[43]. Actually, the CKD-EPI equation seems to be the more precise formula for primary care patients [31], [44]–[47]. However, it is not yet recognized as the gold standard. The third limitation regards the cross-sectional nature of the study, in which only one single measurement per patient has been performed. The absence of a repeated eGFR assessment may potentially have resulted in misclassification of some patients (e.g. of individuals with acute changes in kidney function). In a study conducted by Bottomley et al., the potential overestimation of CKD prevalence after a single eGFR measurement was investigated in 512 factory workers (60.9% males, mean age 43 years) [48]. The repeat analyses conducted 3 months after baseline evaluation revealed no significant change in the mean eGFR. However, 21% of the retested individuals had a change in their category of CKD stage and initial proteinuria was reproducible in only 48% of the cases. In a larger community based study including more than 20,000 patients over 45 years of age, Weiner et al. found that in 76.2% of the patients with initial eGFR <60 ml/min/1.73 m^2^ and in 83.6% of those with eGFR ≥ 60 ml/min/1.73 m^2^ a stable level of renal filtration function was found at follow up [12]. In this report, the study groups had a mean age of 73.4 and 59.9 years, respectively (55% females in both groups) and the follow-up was performed about 3 years after the baseline visit. In these studies, the MDRD estimating equation was used, and the participant’s characteristics were clearly different from our trial. However, the results emphasize the potential of misclassification related to a cross-sectional design. Therefore, it would have been preferable to conduct a longitudinal study with multiple measurements over time to confirm and to adjust the estimated prevalence of CKD and ACR. Moreover, our study excluded paediatric patients, what confines our conclusions to adults. Beside the intrinsic limitations of a cross-sectional design, it is important to remember that the decline of eGFR with ageing is a sign of physiological senescence. With increasing age and consequent decline in muscle mass there is a consecutive reduction in creatinine generation. In the Nijmegen Biomedical Study including about 6,000 apparently healthy persons aged 18–90 years, the eGFR declined approximately 0.4 ml/min/1.73 m^2^ per year [49]. Moreover, an eGFR of 60 ml/min/1.73 m^2^ was within the 25^th^ and 50^th^ percentile for men and women >65 years. In another study including more than 10,000 individuals 66 years of age or older, eGFR reductions of 0.8–1.4 and 2.1–2.7 ml/min/1.73 m^2^ per year were reported for non-diabetic and diabetic subjects, respectively [50]. These data emphasize that a low eGFR in elderly subjects does not necessarily imply that they have kidney disease. Unfortunately, the current NKF-CKD classification does not take into account these aspects. In general, for people with eGFR <60 ml/min/1.73 m^2^ or ACR >30 mg/g (i.e. a suspected CKD) further tests should be performed to determine the type and duration of kidney disease. If the duration is >3 months, CKD is formally established. For elderly patients with slight to moderate reduction of renal function it is particularly important to monitor for rapid progression, defined as a sustained decline in eGFR of more than 5 ml/min/1.73 m^2^ per year [51]. Moreover, some studies have shown that decreased eGFR is independently associated with increased risk of cardiovascular diseases or death [52]. Therefore, a rapid decline in renal function may necessitate an adaptation in treatment strategy. Finally, it is important to note that the majority of previously diagnosed CKD patients, especially those with severe CKD stages requiring dialysis, are usually seen by nephrologists. Therefore, this study should be considered as representative only for adult patients in a primary care setting.

In summary, CKD prevalence in a primary care population in Switzerland is high. The growing proportion of elderly people among the Swiss population and the increasing prevalence of many risk factors will result in an increase of CKD prevalence over the next decades. Implementation of prevention and screening programs will be crucial in the managing strategies of many healthcare systems, especially in western countries. In addition, overcoming the lack of CKD awareness must become part of future strategies. Unlike the United States, where educational efforts have been made to increase CKD awareness in the general population (e.g. the formation of the National Kidney Disease Education Program by the National Institutes of Health), [53] in Switzerland and in many other central European countries, CKD is still an underestimated disease [30]. This study, providing new information on CKD prevalence, may represent a first important step towards challenging this issue. Future steps will be to evaluate the actual burden of CKD, to investigate the prevalence of CKD in an inpatient setting, to model possible trends, and to provide suggestions to avoid uncontrolled growth of the CKD population.
