# Correction: The Prevalence of Chronic Kidney Disease in a Primary Care Setting: A Swiss Cross-Sectional Study

**DOI:** 10.1371/annotation/011969ee-3f4b-4260-8d95-1b9a4ca39008

**Published:** 2013-07-31

**Authors:** Yuki Tomonaga, Lorenz Risch, Thomas D. Szucs, Patrice M. Ambühl

The name of the fourth author was spelled incorrectly. The correct name is: Patrice M. Ambühl. 

The correct citation is: Tomonaga Y, Risch L, Szucs TD, Ambühl PM (2013) The Prevalence of Chronic Kidney Disease in a Primary Care Setting: A Swiss Cross-Sectional Study. PLoS ONE 8(7): e67848. doi:10.1371/journal.pone.0067848.

